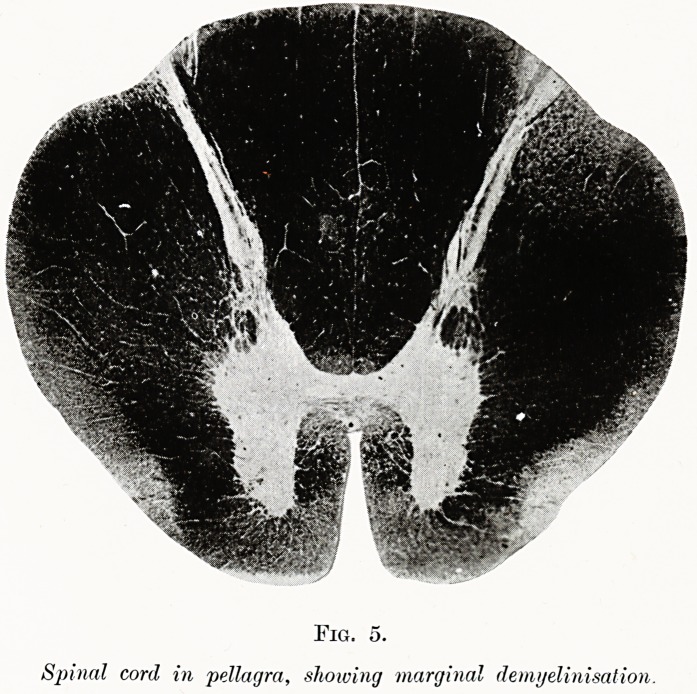# The Central Nervous System in Addisonian Anæmia

**Published:** 1927

**Authors:** Geoffrey Hadfield

**Affiliations:** Pathologist to the General Hospital, Bristol


### THE CENTRAL NERVOUS SYSTEM IN ADDISONIAN ANÆMIA.

BY

GEOFFREY HADFIELD, M.D., M.R.C.P. Lond.,

*Pathologist to the General Hospital, Bristol.*

---

THE typical nervous lesion of pernicious or Addisonian anæmia is sub-acute combined degeneration of the spinal cord, which is accompanied by severe anæmia in so many cases that one synonym for the disease is the "anæmic spinal disease," another "anæmic pseudo-tabes."

The condition is often referred to on the Continent as the syndrome of Lichtheim, who first differentiated it from tabes dorsalis. The term "sub-acute combined degeneration" was first used in 1900 by Russell, Batten and Collier of the National Hospital, Queen Square, in their masterly description of the disease. Four years later Michell Clarke published the results of his extensive experience in an authoritative paper. Following this pioneer work, devoted to establishing the disease on a firmer ætiological and clinical basis, subsequent research, culminating in the work of Price-Jones and Hurst, has pointed, in no uncertain fashion, to the frequent association of neuro-anæmic syndromes with pathological states of the gastro-intestinal tract and notably with achlorhydria, congenital or acquired.

The changes found in the spinal cord post-mortem, although liable to considerable variation in degree, are unique of their kind. When at all well established there is diffuse, non-systematised but roughly symmetrical de-myelinisation and axon-destruction of its least vascular parts, viz. its white matter, the centre of the posterior columns, the marginal zones and the mid-dorsal region. The primary lesion shows no selection of anatomical tracts, and no indication that blood-vessel disease or inflammatory reaction from the meninges or elsewhere initiates the morbid process, whilst a very striking absence of neuroglial scarring makes the disease, in this respect, almost unique. The destructive changes in the white matter are usually slow in evolution, and perhaps occur more frequently than the symptoms suggest.

Starting as microscopic foci of necrosis with a tendency to symmetry, the initial lesions fuse to produce areas of de-myelinisation visible, after suitable staining, to the naked eye, never sharply circumscribed, never invading the grey matter but always retaining their rough symmetry. There is little tendency to shrinkage because of the paucity of neuroglial reaction ; on the other hand, in the early and intermediate stages of the disease there is a tendency to localised swelling from œdema. In one case, where the lesions were discrete, comparatively early and deeply-placed in the posterior columns, the naked-eye appearance of the cord suggested multiple tumour formation. Fig. 1, a low-power micro-photograph of this cord, shows the obvious projection backwards of the posterior columns. In any other type of destructive lesion the tendency would be towards gliosis and shrinkage. When the disease is allowed to run its course the white matter of the mid-dorsal region is eventually destroyed almost completely by the fusion of innumerable foci of necrosis (see Figs. 2 and 3). Finally, secondary ascending and descending tract degeneration, responsible for the terminal flaccid paraplegia, takes place. During the early and intermediate stages, when myelin is being disintegrated, the vessels of the cord frequently show thick cuffs of myelin-phagocytes lying in their perivascular spaces.

Clinically the cases fall into the following three groups, but intermediate types are common :—

1. Cases of typical Addisonian anæmia, in which there is little or no evidence during life that the cord is diseased, but definite though slight evidence of the typical lesion post-mortem.

2. Cases of typical Addisonian anæmia with megalocytosis and mild jaundice, in which clear signs of the spinal cord lesion develop later. In this group half the cases are found.

3. Cases in which there is neither clinical nor numerical anæmia nor any jaundice, but in which neurological signs and symptoms dominate the clinical picture, such cases developing pernicious anæmia either in the late stages of the nervous disease or not at all.

The first group of cases is important chiefly from the ætiological point of view, but it is likely that, if all cases of pernicious anæmia were carefully examined, especially for minor degrees of loss of conduction in the posterior columns and for mild grades of spasticity, the number falling into this group during life would be higher. The percentage of cases of Addisonian anæmia in which the spinal cord is found involved post-mortem is said to lie between 75 and 80 per cent. There seems little doubt that the disease may leave the cord completely untouched. I failed to find any evidence of disease in two cords from typical cases after a long search. Both cases were of a more acute type than the average, and possibly a certain degree of chronicity, especially of the gastro - intestinal symptoms, is necessary before the lesion can be demonstrated by the methods at present at our disposal.

A careful neurological overhaul is of importance in the second group of cases. Here, comparatively early in the course of the anæmia, the patient may complain of numbness and paræsthesiæ of the legs and show a minor degree of loss of the sense of passive movement and position or of vibration sense, before a simple blood examination will provide a definite diagnosis. It would, of course, be one's duty, having discovered such evidence of early and otherwise unexplained spinal cord disease, to eliminate Addisonian anæmia by resorting to an examination of the gastric juice for achlorhydria and to an examination of the blood, with special reference to the mean diameter of the red cells and the detection of hyper-bilirubinæmia, and also of the urine for pathological urobilinuria. At such an early stage the patient may give a history of diarrhœa without obvious cause. The hair is often prematurely grey. Not infrequently the patient gives a clear family history of pernicious anæmia or volunteers the information that he has been noticeably pale for several years, or, in a few cases, actually from childhood.

Those cases in which the gross signs and symptoms of spinal cord disease are present but in which the blood shows no abnormality are not common. Some of these cases present the signs of frank Addisonian anæmia almost as a terminal event. In others no clinical anæmia develops, but examination discloses an increased red cell diameter and perhaps slight jaundice. In a very small number blood changes are absent, and no

PLATE I.

Fig. 1.

*Early lesion in posterior columns: case of scirrhous cancer of stomach. Lesion has produced considerable swelling.*

(*Weigert-Pal preparation.*)

Fig. 2.

*Sub-acute combined degeneration (advanced).*

*Foci of degeneration are pale.*

Fig. 3.

*Normal cord stained with above, for comparison.*

PLATE II.

Fig. 4.

*Sub-acute combined degeneration occurring in connection with scirrhous cancer of the stomach. (Weigert-Pal.)*

Fig. 5.

*Spinal cord in pellagra, showing marginal demyelinisation.*

signs of increased hæmolysis can be made out. In some of these achlorhydria is present and, perhaps, when kept under observation for long periods, several slight and almost unnoticed attacks or one or more severe bouts of hæmolytic anæmia may appear.

About twelve months ago I saw with Dr. McKenzie of Southville a middle-aged, grey-haired man in the early stages of subacute combined degeneration of the cord whose blood was normal in every respect, and in whom no evidence of increased internal hæmolysis could be found. He had a total achlorhydria. A short time ago I again measured his mean red cell diameter and found it normal; he has now undoubted subacute combined degeneration.

In such cases the problem for solution is whether subacute combined degeneration ever develops apart from hæmolytic anæmia. We cannot accept this as proved in any given case unless it has been observed long enough to exclude the occurrence of hæmolytic periods, either intercurrent or terminal.

In considering these three clinical groups there seems, therefore, to be ample evidence for assuming that the central nervous system in Addisonian anæmia may suffer attacks of every degree of severity, in the majority of cases apparently escaping altogether, in others showing lesions which require care and microscopic vision for their detection; in some cases the cord is involved late in the disease, whilst in others it bears the brunt of the attack, no anæmia capable of giving rise to symptoms or serious visceral change developing. There is a similar variation in the duration of the disease, in the intensity of the diarrhœa, in the degree of hæmolysis and in the soreness of the tongue.

Clinically, and from the neurological point of view, the symptoms and signs of the nervous lesion vary in intensity and duration within very wide limits. The course of the average typical case is usually divisible into three stages :—

1. A *Prodromal Stage* of four to twelve months characterised by subjective sensations in the limbs, mild spasticity and alteration in conduction of deep sensation.

2. The *Stage of Onset*, in which either ataxia from loss of deep sensation, spastic paraplegia with increased deep reflexes from pyramidal disease, or a combination of ataxia and spasticity, gradually evolves over a period of several months.

3. The *Terminal Stage*, in which the paraplegia becomes flaccid with loss of reflexes and superficial sensation becomes profoundly altered. The legs become œdematous, the sphincters fail, and there are frequently mental changes. The average course of the disease is under two years, and it is invariably fatal. The subjective sensations observed by the patient are usually the first signs of the disease, and take the form of numbness, tingling or pricking sensations in the limbs and sensations of cold and heat. Symptoms such as these in a patient between 45 and 50 call urgently for a blood examination. During this stage, unless prevented by the anæmia, the patient is usually working and walking. Sooner or later the majority of patients show impairment in conduction of deep sensation, especially the sense of passive movement and position and vibration sense, or, if the pyramidal tracts are picked out, weakness and mild spasticity with increased tendon jerks. For four to twelve months these symptoms slowly increase in intensity and insensibly there develops either a gross ataxia, a definite paraplegia or a mixture of pyramidal and posterior column disease. On the other hand, a patient who has been in the prodromal stage for a couple of months may be suddenly precipitated in a few days or even in a night into spastic paraplegia or severe ataxia.

Much the same degree of variation characterises the transition between the stage of the onset and the terminal stage, the first sign of which is usually weakening of the ankle jerk. The terminal stage in quiet cases may last six months, involve the trunk and lead to muscular atrophy of the legs and hands.

Finally, there remains the fundamental problem of the causation of the nervous lesions, and at the outset we must decide whether similar lesions occur in other diseases. If we take the broader characteristics of this affection—the diffuse character of the non-systematised and roughly symmetrical destruction of the least vascular parts of the spinal cord—and disregard histological minutiæ and variations in the healing process, we find that similar lesions, although uncommon, occur in association with a series of conditions in all of which there is a strong probability of chronic toxic absorption from the stomach and small bowel. Following the long-continued achlorhydria of the more chronic types of cancer of the stomach, a lesion, essentially similar to that of subacute combined degeneration of the spinal cord, is occasionally found. In a few of these cases a hæmolytic anæmia with increase in the average size of the red corpuscles and deposition of iron in the liver may develop, giving a clinical picture very like that of pernicious anæmia, except that there is some degree of cancerous cachexia. It seems very probable that in these cases the gastric neoplasm sets up conditions necessary for the development of a toxic hæmolytic anæmia approximating to the pernicious type, including an initial achlorhydria.

I investigated such a case for Dr. Carey Coombs in 1923. The patient, a wasted, slightly jaundiced and very anæmic man of 54, complained of tingling of the legs and difficulty in walking upstairs. His blood showed profound anæmia with a high colour index ; and post-mortem his viscera showed moderate siderosis. Lying in the greater curvature of the stomach was a dense spindle-shaped mass almost cartilaginous in consistence and about 1½ inches in its longest diameter. On section the mass proved to be an adeno-carcinoma with a dense fibrotic stroma. The white matter of the spinal cord showed non-systematised degeneration in a comparatively early phase (see Fig. 4). The sequence of the events in this case would appear to be a primary scirrhous carcinoma of the stomach with achlorhydria, followed by a secondary hæmolytic anæmia and subacute combined degeneration of the cord. Very closely allied to such a case are those in which subacute combined degeneration has followed gastrectomy.

A lesion of the cord very similar in many ways to that of Addisonian anæmia is also found in practically all fatal cases of pellagra, in which bouts of diarrhœa occur as a prominent symptom during life, and atrophy of the small bowel is discovered post-mortem. I have investigated a typical case with Dr. Barton White of the Bristol Mental Hospital, in which marginal destruction was present on both sides of the cord, together with striking naked-eye atrophy and thinning of the wall of the small bowel (see Fig. 5). In this connection it is interesting to note that when gastrectomy is survived for a long period pellagra may subsequently develop in the absence of the usual dietetic factors. An account of another case, also studied at the Bristol Mental Hospital by Dr. Barton White and myself, will be found in the next paper of the present issue.

That there is probably a lymphatic pathway capable of transporting bacterial toxins from the peritoneal cavity to the spinal cord, and that such toxins may produce lesions similar to those of subacute combined degeneration, is strongly suggested by the work of Orr and Rows, who introduced celloidin capsules containing living staphylococci into the peritoneal cavity of rabbits and found widespread degeneration of the white matter of the cord similar in type to that occurring in Addisonian anæmia.

In conclusion, therefore, we may say that subacute combined degeneration of the cord is not confined to cases of Addisonian anæmia, nor is it diagnostic of this. Whenever it is found there has usually preceded it a long period during which gastro-intestinal symptoms were present and toxic absorption from the small bowel probable. The spinal cord lesion differs in several essentials from that found in other degenerative conditions, so much so that it is almost unique. There is a strong probability that the lesion is toxic in nature and the toxin intestinal in origin and bacterial in type, a conclusion supported by the demonstration in animals of a free lymphatic pathway from the peritoneal cavity to the spinal cord, and by the production of essentially similar experimental lesions by bacterial toxins.

As a working hypothesis it is not unreasonable to incriminate congenital or acquired achlorhydria as the primary and central ætiological factor in the production of subacute combined degeneration. From the clinical point of view, a complete neurological overhaul especially directed to discover loss of conduction of deep sensation or the presence of mild degrees of pyramidal involvement will in some cases lead one to a diagnosis of Addisonian anæmia, whilst in others it may help considerably in confirming such a diagnosis in doubtful or early cases.

## Figures and Tables

**Fig. 1. f1:**
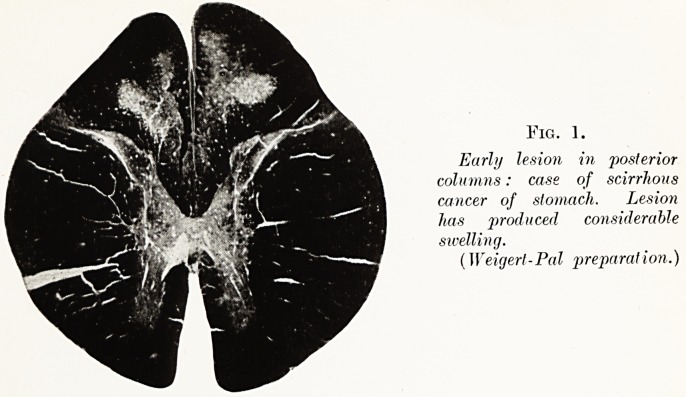


**Fig. 2. f2:**
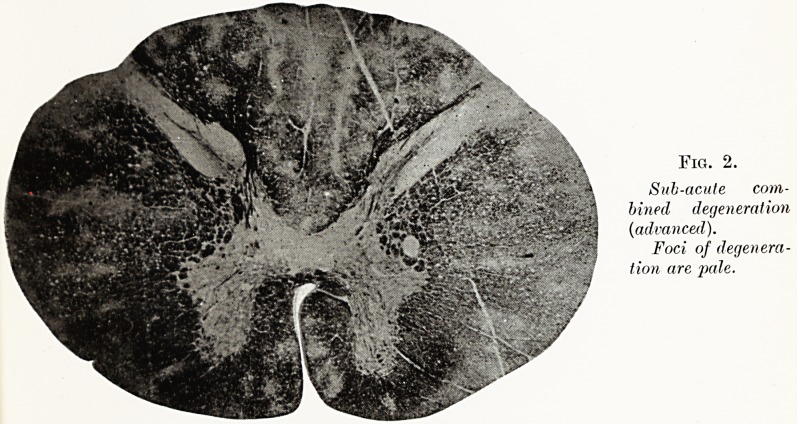


**Fig. 3. f3:**
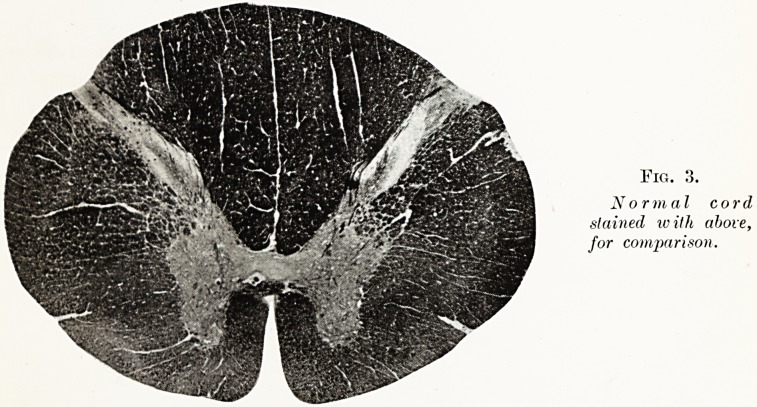


**Fig. 4. f4:**
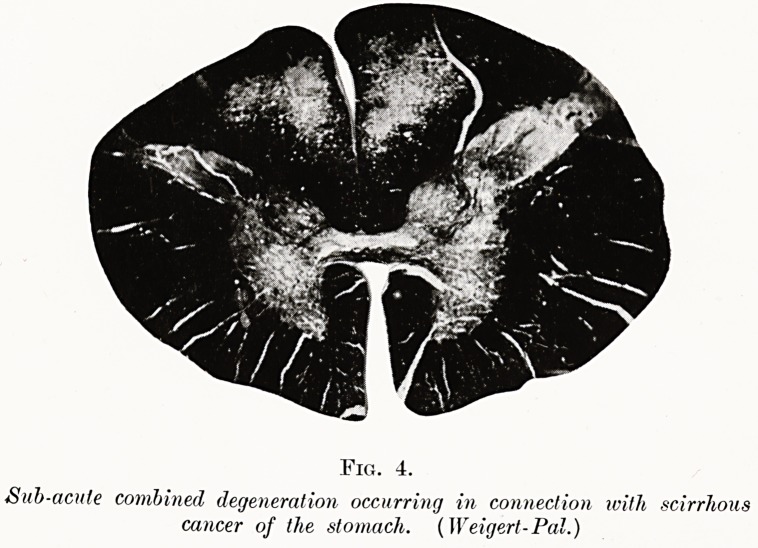


**Fig. 5. f5:**